# Associations of body shapes with insulin resistance and cardiometabolic risk in middle-aged and elderly Chinese

**DOI:** 10.1186/s12986-021-00629-1

**Published:** 2021-12-07

**Authors:** Yulin Zhou, Yanan Hou, Jiali Xiang, Huajie Dai, Mian Li, Tiange Wang, Shuangyuan Wang, Hong Lin, Jieli Lu, Yu Xu, Yuhong Chen, Weiqing Wang, Yufang Bi, Min Xu, Zhiyun Zhao

**Affiliations:** 1 Department of Endocrine and Metabolic Diseases, Shanghai Institute of Endocrine and Metabolic Diseases, Ruijin Hospital, Shanghai Jiao Tong University School of Medicine, 197 Ruijin 2nd Road, 200025 Shanghai, China; 2 Shanghai National Clinical Research Center for Metabolic Diseases, Key Laboratory for Endocrine and Metabolic Diseases of the National Health Commission of the PR China, Shanghai National Center for Translational Medicine, Ruijin Hospital, Shanghai Jiao Tong University School of Medicine, Shanghai, China

**Keywords:** Body shape, Anthropometric traits, Insulin resistance, Cardiometabolic disorders

## Abstract

**Background:**

We aimed to define refined body shapes by using multiple anthropometric traits that represent fat distribution, and evaluate their associations with risk of insulin resistance (IR) and cardiometabolic disorders in a Chinese population.

**Methods:**

We performed a cross-sectional analysis in 6570 community-based participants aged ≥ 40 years. Four body circumferences (neck, waist, hip, and thigh) and their ratios were put simultaneously into an open-source Waikato Environment for Knowledge Analysis platform to select the worthiest indicators in determining IR. The ratio of the top 3 fat distribution indicators was used to define the refined body shapes.

**Results:**

We defined 8 distinct body shapes based on sex-specific combinations of waist-to-hip ratio (WHR), waist-to-thigh ratio (WTR), and waist-to-neck ratio (WNR), which differed in participants’ distribution and risk of IR and related cardiometabolic disorders. In women, as compared to the low WHR-low WTR-low WNR shape, all body shapes were significantly associated with IR and related cardiometabolic disorders; while in men, the low WHR-high WTR-high WNR shape and the higher WHR related shapes were significantly associated with IR and related cardiometabolic disorders. Stratified by WHR, the results were consistent in women; however, no significant associations were detected in men.

**Conclusions:**

We defined 8 distinct body shapes by taking WHR, WTR, and WNR, simultaneously into account, which differed in association with the risk of IR and related cardiometabolic disorders in women. This study suggests that body shapes defined by multiple anthropometric traits could provide a useful, convenient, and easily available method for identifying cardiometabolic risk.

**Supplementary Information:**

The online version contains supplementary material available at 10.1186/s12986-021-00629-1.

## Background

Obesity is a global public health concern. More than 1 billion adults suffer from overweight, and 650 million adults and 124 million adolescents suffer from obesity worldwide [1]. Obesity and related cardiometabolic disorders, such as insulin resistance, metabolic syndrome, hyperlipidemia, etc., are responsible for type 2 diabetes, cancers, cardiovascular diseases and related mortality [2–4]. To better identify and define obesity is extremely essential for the prevention and management of comorbidity and mortality.

Anthropometric traits such as body mass index (BMI), waist circumference, and waist-to-hip ratio (WHR) were widely used to classify obesity and body shapes (the apple or pear type), which plays a vital role in evaluating cardiometabolic risk [5–7]. However, these metrics cannot adequately discriminate variation in fat distribution and cannot adequately evaluate individual cardiometabolic risk. In clinical practice, individuals with the same BMI or waist circumference may have different health conditions. Some obese individual has been considered as “metabolically healthy obese [8], and some individuals in a normal range of BMI, still at increased risk of type 2 diabetes [9], suggesting that other factors, including other ectopic fat depots, may have a contribution [10].

Integrating multiple anthropometric information aids in defining a more accurate cardiometabolic risk [11–15]. For example, when incorporated calf circumference into the definition of metabolic syndrome, it demonstrated a higher cardiovascular and all-cause mortality risk than the traditional definition of metabolic syndrome alone [14]. Most indices of central fatness, including waist circumference, WHR, body adiposity index and a body shape index, were positively associated with a higher all-cause mortality risk independent of overall adiposity, which indicated measures of central adiposity could be used as a supplementary approach, in combination with BMI, to determine the risk of premature death [15]. Upper body subcutaneous fat is a distinct fat depot separate from abdominal subcutaneous fat that may confer increased cardiometabolic risk [16]. Neck circumference, as an indirect measure of upper body subcutaneous fat, is associated with cardiometabolic risk factors and subclinical atherosclerosis independent of BMI [16]. These previous studies suggested novel anthropometric traits play a vital role in predicting cardiometabolic risk.

In addition, it’s uncertain whether the combination of multiple anthropometric traits may identify additional risk assessment of cardiometabolic disorders. Therefore, our study aimed, firstly, to define body shapes in a sex-specific manner by using several body circumferences or their ratio; secondly, to evaluate their associations with risk of insulin resistance and several major cardiometabolic disorders. The study will provide refined body shapes and further stratify individuals into different stratification of cardiometabolic risk based on these distinct body shapes.

## Methods

### Study population

The current cross-sectional analysis was based on one of the follow-up circles of our previous community-based cohort studies [17, 18]. Briefly, between August 2014 and May 2015, 6570 registered permanent residents aged ≥ 40 years from Jiading district, Shanghai, China, participated in the health examination aimed to explore the effects of risk factors on type 2 diabetes and related chronic diseases. Participants missing data on anthropometric traits including height, weight, waist circumference, hip circumference, thigh circumference and neck circumference (n = 167), or missing data on biochemical measurements including systolic and diastolic blood pressure, fasting and 2 h-OGTT plasma glucose, fasting serum insulin, total cholesterol, triglycerides, high-density lipoprotein cholesterol and low-density lipoprotein cholesterol (n = 163) were excluded. Finally, 6240 participants were included in the current analysis.

The study protocol was approved by the Institutional Review Board of Ruijin Hospital affiliated to Shanghai Jiao Tong University School of Medicine. All participants consented to the study and signed informed consent.

### Anthropometric measurements

A standard questionnaire was used to collect information on sociodemographic characteristics, chronic diseases and medical history, physical activity and lifestyle factors (e.g. smoking and alcohol status). If participants consumed any kinds of cigarettes or alcohol regularly in the past 6 months, the current smoking or drinking status was defined as ‘yes’. Physical activity in terms of MET hour/week was acquired and calculated according to the short form of the International Physical Activity Questionnaire (IPAQ) [19].

Anthropometric traits including height, weight, neck circumference, waist circumference, hip circumference and thigh circumference were measured by well-trained physicians according to a standard protocol. BMI was calculated as body weight in kilograms divided by body height squared in meters (kg/m^2^). Waist circumference was measured at the level of the umbilicus with the patient in the standing position. Hip circumference was measured at the tip of the bone around the greater trochanter of the femur. Thigh circumference was measured on the right leg directly below the gluteal fold [20]. Neck circumference was measured in the midway of the neck, below the laryngeal prominence [21]. Systolic and diastolic blood pressure were measured at the non-dominant arm with an automated electronic device (OMRON Model HEM-752 FUZZY, Omron Company, Dalian, China) three times consecutively with a 1-min interval after at least 10-min rest in the seated position. The average value of the three measurements was used in our analysis.

### Biochemical measurements

All participants underwent standard 75-g oral glucose tolerance tests (OGTT) after overnight fasting of more than 10 h. Fasting and 2 h-OGTT plasma glucose concentrations were measured using the glucose oxidase method through an autoanalyzer (Modular P800; Roche, Basel, Switzerland). Fasting and 2 h-OGTT serum insulin concentrations, and serum lipid profiles including total cholesterol, triglycerides, high-density lipoprotein (HDL) cholesterol and low-density lipoprotein (LDL) cholesterol were measured using an electrochemiluminescence assay (Modular E170; Roche, Basel, Switzerland).

### Definitions

Insulin resistance index (homeostasis model assessment of insulin resistance, HOMA-IR) was calculated as fasting serum insulin (μIU/mL) × fasting plasma glucose (mmol/L) / 22.5. Excluding individuals treated with insulin or hypoglycemic agents, insulin resistance was defined as HOMA-IR ≥ 2.61, which was the highest quartile of HOMA-IR. Diabetes was defined as fasting plasma glucose ≥ 7.0 mmol/L or 2 h-OGTT plasma glucose ≥ 11.1 mmol/L or use of antidiabetic agents. Metabolic syndrome was diagnosed based on the National Cholesterol Education Program Adult Treatment Panel III criteria [22]. Individuals with three or more of the following five components were diagnosed to have metabolic syndrome: (1) blood pressure ≥ 130/85 mmHg or taking antihypertensive drugs; (2) waist circumference ≥ 88 cm for women (≥ 102 cm for men); (3) serum triglyceride level ≥ 1.7 mmol/L; (4) serum HDL cholesterol level < 1.29 mmol/L for women (< 1.04 for men); (5) fasting plasma glucose level ≥ 6.1 mmol/L or confirmed diagnosis of diabetes.

### Definition of body shapes

Automated feature selection was performed by using the information gain attribute ranking method on the open-source Waikato Environment for Knowledge Analysis platform [23]. Information gain ranking was used to evaluate the worth of each variable (usually the clinical indicator) by measuring the entropy gain to the outcome. The greater the information gain a clinical indicator has, the more important the indicator is in the classification process. The model was built with logistic regression analysis in a sex-specific manner. The regression coefficient of each significant variable was regarded as the contribution level (Additional file 1: Fig. S1A) [23].

In the present study, we used insulin resistance as the outcome to evaluate the potential dominant anthropometric traits in a sex-specific manner. Anthropometric traits including BMI, WHR, waist-to-thigh ratio (WTR), waist-to-neck ratio (WNR), neck-to-thigh ratio, neck-to-hip ratio, thigh-to-hip ratio were put into the selection process. As a result, BMI ranked first for determining insulin resistance, followed by WHR, WTR, and WNR in both men and women (Additional file 1: Fig. 1B).

**Fig. 1:**
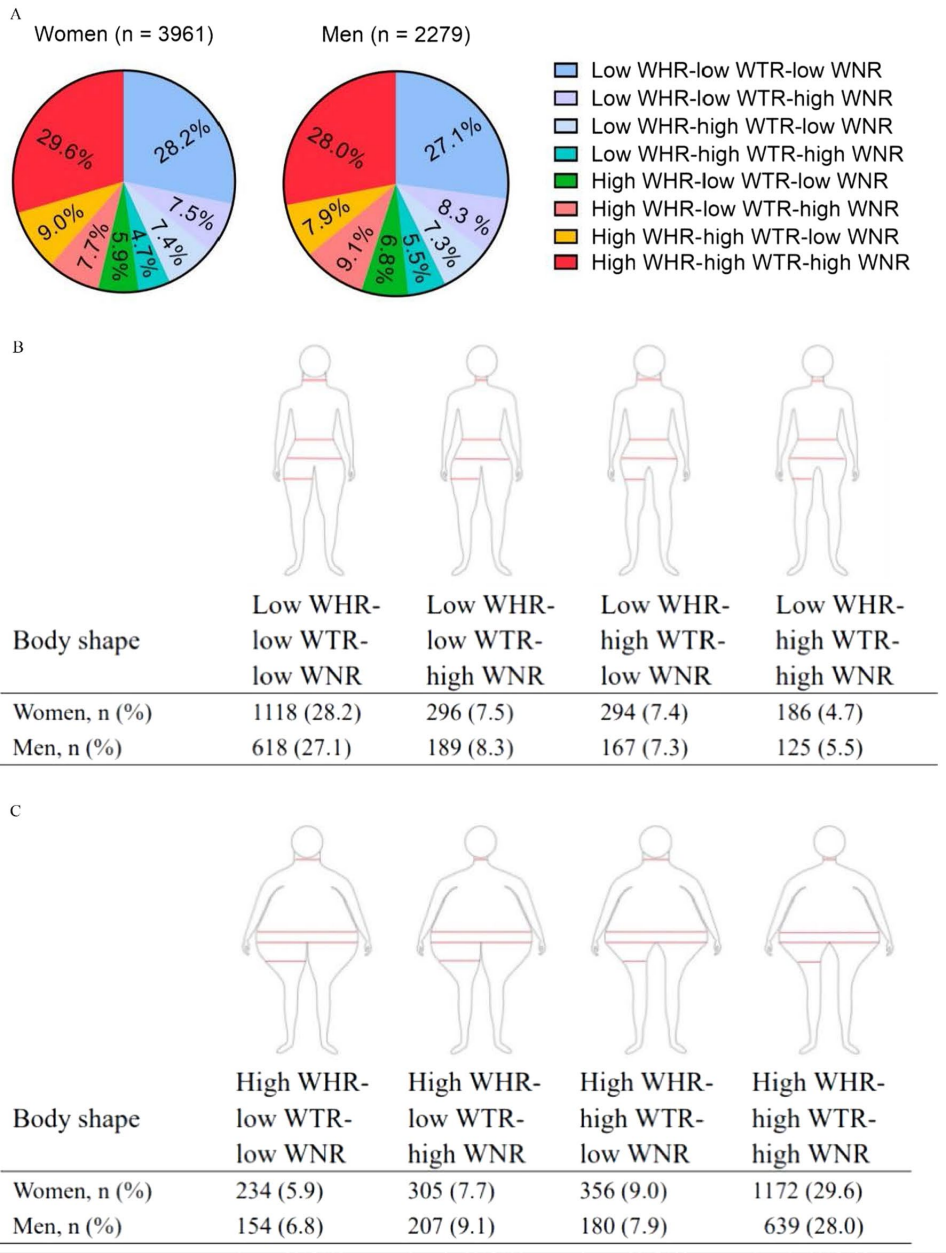
The distribution of body shapes in sex-specific groups. The individuals were divided into high- and low-level groups according to the medians of WHR, WTR, and WNR, respectively. Based on the 3 derived ratios, WHR, WTR, and WNR, we defined 8 distinct body shapes. The corresponding median value for WHR is 0.87 in women and 0.91 in men, for WTR is 1.66 in women and 1.74 in men, and for WNR is 2.48 in women and 2.34 in men. Abbreviation: WHR, waist-to-hip ratio; WTR, waist-to-thigh ratio; WNR, waist-to-neck ratio

As expected, BMI ranked first for determining insulin resistance in our analysis. According to our study objective, we aimed to define body shapes by using several circumferences or their ratios to identify additional risk assessment. Therefore, we took the 3 following ratios, WHR, WTR, and WNR, simultaneously into account to define the body shapes in a sex-specific manner and evaluate their associations with risks of insulin resistance and cardiometabolic disorders.

We calculated the medians of these 3 traits in a sex-specifically manner. The corresponding median value for WHR was 0.87 in women and 0.91 in men, for WTR was 1.66 in women and 1.74 in men, and for WNR was 2.48 in women and 2.34 in men. We performed a receiver operating characteristic (ROC) scatter plot analysis to examine the best cut-off value of WHR, WTR and WNR in relation to insulin resistance. We found that the best cut-off value of these 3 metrics almost equal to the median values. According to the medians of WHR, WTR, and WNR, individuals were divided into the high- and low-level groups respectively. Based on the 3 derived ratios, WHR, WTR, and WNR, we defined 8 distinct body shapes (Fig. 1).

### Statistical analysis

Data were summarized as means ± standard deviation (SD) or median (inter-quartile ranges) for continuous variables, and numbers (percentages) for categorical variables. Multiple variable logistic regression analysis was performed to evaluate associations of body shapes with insulin resistance and cardiometabolic disorders in sex-specific samples. Odds ratios (ORs) and the corresponding 95% confidence intervals (CI) were calculated. The adjustments included age (years), BMI (kg/m^2^), current smoking (yes or no), current drinking (yes or no) and physical activity (MET-h/wk).

To assess the added value of the defined body shape in predictive models, we included these body shapes in the models of predicting cardiometabolic risk. We calculated the difference with or without the defined body shape (ΔC statistic), net reclassification improvement (NRI), and integrated discrimination improvement (IDI). The C statistic measures concordance between model-based risk estimates and observed events. NRI and IDI measure the incremental prognostic effect that a new biomarker will have when added to an existing prediction model.

All the statistical analyses were performed with SAS version 9.4 (SAS Institute Inc, Cary, NC, USA). Statistical significance was set to a two-sided *P* value < 0.05.

## Results

### Characteristics of study participants

The present study including 3961 (63.4%) women and 2279 (36.3%) men with an average BMI was 25.0 ± 5.9 kg/m^2^ for women and 25.1 ± 3.5 kg/m^2^ for men. Compared with men, women were younger, had lower level of several major anthropometric traits including neck circumference, waist circumference, hip circumference, thigh circumference, WHR, WTR, neck-to-hip ratio, and neck-to-thigh ratio; higher level of HOMA-IR, systolic blood pressure, LDL- and HDL cholesterol and triglycerides (All *P* < 0.05, Table 1).

**Table 1:** Characteristics of study participants in sex-specific samples

	Total	Women		Men		*P* value
	/	/	Range	/	Range	
n (%)	6240	3961 (63.4)	/	2279 (36.3)	/	/
Age (years)	62.2 ± 8.74	61.9 ± 8.56	44.3–91.2	62.9 ± 9.03	40.1–94.9	< 0.0001
Systolic blood pressure (mmHg)	134.8 ± 17.3	135.1 ± 17.7	66.3–224.7	134.2 ± 16.5	89.0–213.3	0.03
Diastolic blood pressure (mmHg)	76.3 ± 9.5	75.4 ± 9.5	42.0–127.3	77.8 ± 9.7	42.3–124.7	< 0.0001
Current smoking, n (%)	1224 (19.2)	20 (0.5)	/	1204 (51.5)	/	< 0.0001
Current drinking, n (%)	900 (14.1)	45 (1.11)	/	855 (36.5)	/	< 0.0001
Physical activity (MET-h/wk)	18 (4.5–21.0)	21.0 (6.0–21.0)	0–2380	15.0 (0.0–21.0)	0–489	0.60
*Biochemical measurements*						
Fasting plasma glucose (mmol/L)	6.15 ± 1.47	6.06 ± 1.34	1.40–20.8	6.29 ± 1.66	4.21–20.0	< 0.0001
HOMA-IR	1.89 (1.27–2.83)	1.97 (1.34–2.91)	0.13–224	1.74 (1.13–2.61)	0.19–226	< 0.0001
Total cholesterol (mmol/L)	5.95 ± 0.96	6.54 ± 0.96	2.44–19.3	4.95 ± 0.89	2.68–8.42	0.26
LDL cholesterol (mmol/L)	3.67 ± 4.13	3.83 ± 5.16	1.51–10.9	3.39 ± 0.71	1.14–6.07	< 0.0001
HDL cholesterol (mmol/L)	1.34 ± 0.29	1.39 ± 0.30	0.60–3.28	1.25 ± 0.29	0.68–3.08	< 0.0001
Triglycerides (mmol/L)	1.51 (1.09–2.14)	1.55 (1.12–2.19)	0.45–46.2	1.43 (1.03–2.04)	0.38–15.96	< 0.0001
*Anthropometric measurements*						
Body mass index (kg/m^2^)	25.0 ± 5.2	25.0 ± 5.9	15.0–35.1	25.1 ± 3.5	17.6–37.2	0.41
Waist circumference (cm)	83.6 ± 9.9	81.8 ± 10.0	30.0–163.0	86.7 ± 9.8	35.0–164.0	< 0.0001
Neck circumference (cm)	34.4 ± 3.2	33.0 ± 3.2	18.4–79.0	36.9 ± 3.3	25.2–70.0	< 0.0001
Hip circumference (cm)	93.9 ± 7.7	93.2 ± 8.0	25.0–201.0	95.0 ± 7.1	29.3–166.2	< 0.0001
Thigh circumference (cm)	49.2 ± 5.4	48.9 ± 5.4	16.0–105.0	49.8 ± 5.3	24.6–99.2	< 0.0001
Waist-to-hip ratio	0.89 ± 0.11	0.88 ± 0.12	0.31–3.00	0.91 ± 0.09	0.39–2.39	< 0.0001
Neck-to-hip ratio	0.37 ± 0.05	0.36 ± 0.05	0.17–1.30	0.39 ± 0.04	0.24–1.24	< 0.0001
Waist-to-thigh ratio	1.71 ± 0.22	1.69 ± 0.23	0.58–4.91	1.75 ± 0.21	0.69–3.77	< 0.0001
Neck-to-thigh ratio	0.71 ± 0.09	0.68 ± 0.09	0.32–2.67	0.75 ± 0.09	0.40–2.14	< 0.0001
Waist-to-neck ratio	2.43 ± 0.24	2.48 ± 0.25	0.40–4.97	2.35 ± 0.23	1.00–4.08	< 0.0001
Hip-to-thigh ratio	1.92 ± 0.21	1.92 ± 0.22	0.54–6.03	1.92 ± 0.20	0.51–4.16	0.82
*Cardiometabolic disorders*						
Insulin resistance, n (%)	1814 (29.1)	1246 (31.5)	/	568 (24.9)	/	< 0.0001
Metabolic syndrome, n (%)	2199 (35.2)	1614 (40.8)	/	585 (26.7)	/	< 0.0001
Elevated fasting blood glucose, including diabetes, n (%)	2423 (38.8)	1467 (37.0)	/	956 (42.0)	/	0.0001
High blood pressure, n (%)	4424 (70.9)	2795 (70.6)	/	1629 (71.5)	/	0.44
High triglyceride, n (%)	2580 (41.4)	1723 (43.5)	/	857 (37.6)	/	< 0.0001
Low HDL cholesterol, n (%)	2102 (33.7)	1595 (40.3)	/	507 (22.3)	/	< 0.0001

Data are presented as means ± standard deviation (SD), or medians (inter-quartile ranges) for skewed variables, or numbers (proportions) for categorical variables. *P* values were calculated from one-way analysis of variance (ANOVA) for continuous variables and chi-square test for categorical variables. Insulin resistance was defined as HOMA-IR ≥ 2.61, which is the cut-off point for the highest quartile of the total participants

MET, metabolic equivalent task; HOMA-IR indicates homeostasis model assessment of insulin resistance; LDL cholesterol, low-density lipoprotein cholesterol; HDL cholesterol, high-density lipoprotein cholesterol

### Distribution of body shapes

The 8 distinct body shapes and their proportions by sex were shown in Fig. 1. 29.6% of women and 28.0% of men were assigned to the high WHR-high WTR-high WNR shape, 28.2% of women and 27.1% of men were the low WHR-low WTR-low WNR shape. Few individuals were assigned to the low WHR-high WTR-high WNR shape (women vs. men: 4.7% vs. 5.5%) or the high WHR-low WTR-low WNR shape (women vs. men: 5.9% vs. 6.8%). The proportions of other body shapes varied from 7.3 to 9.1%.

### Body shape in relation to insulin resistance and cardiometabolic disorders

**Fig. 2:**
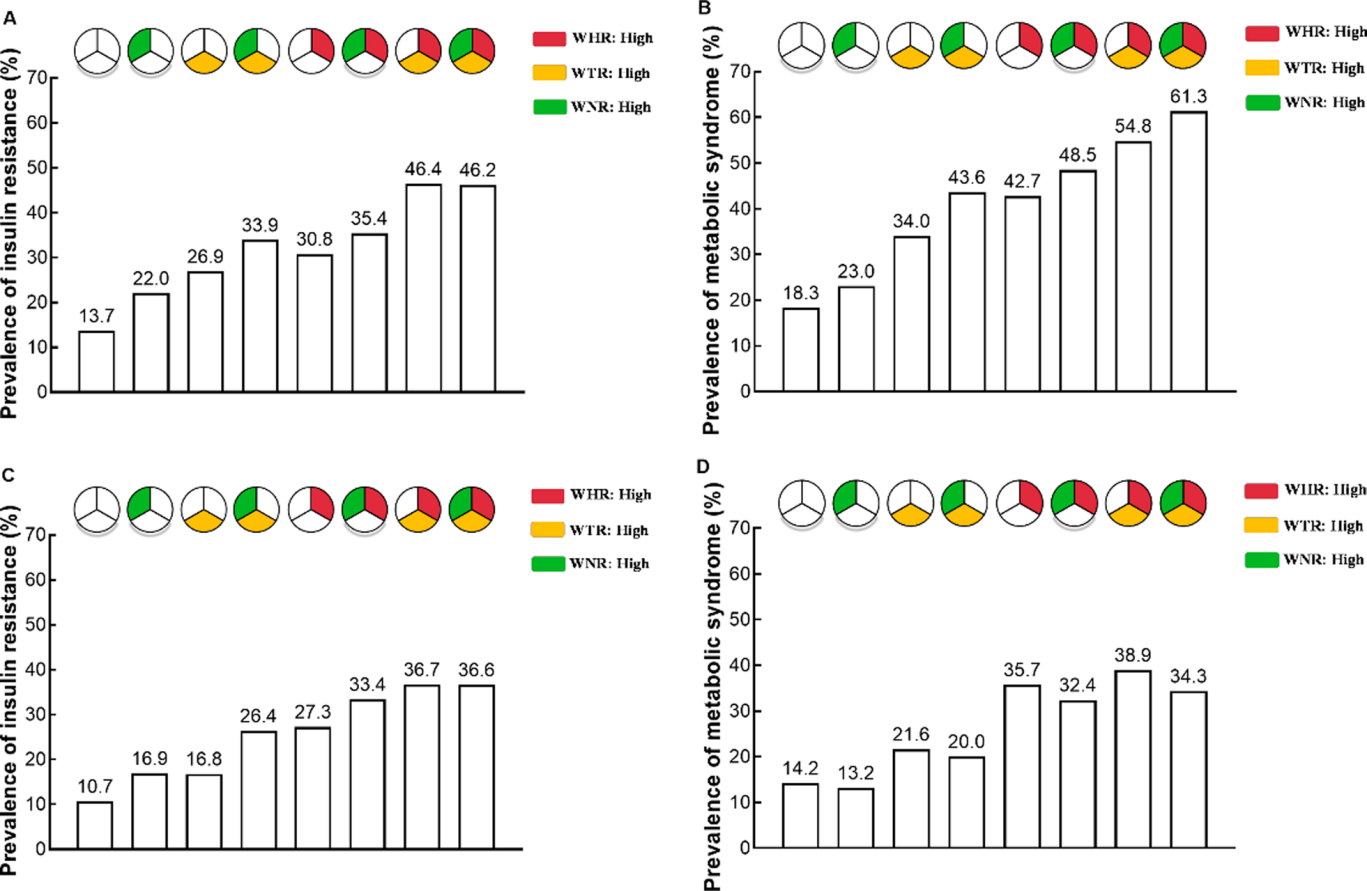
The prevalence of insulin resistance and metabolic syndrome according to 8 body shapes by sex. **A** The prevalence of insulin resistance in women. **B** The prevalence of metabolic syndrome in women. **C** The prevalence of insulin resistance in men. **D** The prevalence of metabolic syndrome in men. WHR, waist-to-hip ratio; WTR, waist-to-thigh ratio; WNR, waist-to-neck ratio

The prevalence of insulin resistance and metabolic syndrome according to the 8 body shapes by sex were shown in Fig. 2. In women, as compared to low WHR-low WTR-low WNR shape, all body shapes were positively associated with insulin resistance. These associations were strongest for the high WHR-high WTR-low WNR shape (OR [95% CI] was 3.71 [2.79–4.93], *P* < 0.0001, Table 2). In men, as compared to low WHR-low WTR-low WNR shape, low WHR-high WTR-high WNR shape and the higher WHR related shapes were positively associated with insulin resistance. These associations were strongest for the high WHR-high WTR-low WNR shape (OR [95% CI] was 3.00 [1.98–4.55], *P* < 0.0001, Table 2).

**Table 2:** Association of body shape with risk of cardiovascular diseases in sex-specific samples

Body shape	Insulin resistance	Metabolic syndrome	Elevated fasting blood glucose, including diabetes	High blood pressure	High triglycerides	Low HDL cholesterol
*Women*						
Low WHR-low WTR-low WNR	Ref	Ref	Ref	Ref	Ref	Ref
Low WHR-low WTR-high WNR	1.49 (0.89–1.75)	0.96 (0.70–1.33)	0.99 (0.74–1.34)	0.85 (0.65–1.12)	1.41 (1.08–1.85)*	1.09 (0.83–1.44)
Low WHR-high WTR-low WNR	2.09 (1.51–2.88)*	1.97 (1.47–2.65)*	1.77 (1.34–2.33)*	1.59 (1.18–2.14)*	1.57 (1.20–2.05)*	1.79 (1.37–2.33)*
Low WHR-high WTR-high WNR	2.09 (1.44–3.02)*	2.17 (1.53–3.06)*	2.32 (1.67–3.23)*	1.27 (0.88–1.84)	1.90 (1.38–2.63)*	1.15 (0.82–1.61)
High WHR-low WTR-low WNR	2.02 (1.44–2.84)*	2.57 (1.89–3.51)*	1.31 (0.96–1.80)	1.54 (1.13–2.12)*	2.26 (1.69–3.01)*	2.14 (1.60–2.86)*
High WHR-low WTR-high WNR	1.92 (1.41–2.61)*	2.59 (1.94–3.45)*	1.37 (1.03–1.83)*	1.78 (1.31–2.40)*	2.25 (1.73–2.93)*	1.77 (1.36–2.31)*
High WHR-high WTR-low WNR	3.71 (2.79–4.93)*	3.57 (2.72–4.69)*	2.36 (1.82–3.06)*	2.23 (1.63–3.06)*	3.15 (2.44–4.06)*	2.13 (1.65–2.74)*
High WHR-high WTR-high WNR	2.84 (2.25–3.58)*	3.81 (3.08–4.71)*	1.94 (1.59–2.38)*	1.85 (1.49–2.30)*	2.33 (1.93–2.82)*	1.66 (1.37–2.01)*
*Men*						
Low WHR-low WTR-low WNR	Ref	Ref	Ref	Ref	Ref	Ref
Low WHR-low WTR-high WNR	1.23 (0.76–1.98)	0.67 (0.41–1.10)	0.73 (0.51–1.05)	0.78 (0.55–1.11)	1.09 (0.75–1.59)	0.97 (0.62–1.53)
Low WHR-high WTR-low WNR	1.49 (0.91–2.44)	1.50 (0.96–2.34)	1.30 (0.91–1.85)	1.08 (0.74–1.58)	1.85 (1.27–2.69)*	1.30 (0.83–2.04)
Low WHR-high WTR-high WNR	2.41 (1.48–3.93)*	1.20 (0.73–2.00)	1.25 (0.84–1.87)	1.00 (0.65–1.54)	1.28 (0.82–1.98)	1.04 (0.61–1.77)
High WHR-low WTR-low WNR	2.11 (1.38–3.32)*	2.32 (1.54–3.52)*	1.83 (1.27–2.64)*	1.29 (0.86–1.93)	2.48 (1.70–3.63)*	2.02 (1.33–3.06)*
High WHR-low WTR-high WNR	2.11 (1.39–3.20)*	1.61 (1.09–2.39)*	1.37 (0.98–1.92)	1.01 (0.70–1.47)	2.43 (1.71–3.46)*	2.11 (1.44–3.11)*
High WHR-high WTR-low WNR	3.00 (1.98–4.55)*	2.48 (1.67–3.67)*	1.81 (1.28–2.57)*	2.01 (1.30–3.11)*	2.48 (1.73–3.57)*	2.00 (1.34–2.99)*
High WHR-high WTR-high WNR	2.62 (1.87–3.66)*	1.80 (1.32–2.46)*	1.51 (1.17–1.94)*	1.70 (1.26–2.28)*	1.89 (1.44–2.48)*	1.43 (1.04–1.96)*

Data were presented as odds ratio (OR) and 95% confidence interval (CI). *P* values were calculated from multivariable logistic regression analysis. Adjusted age (years), body mass index (kg/m^2^), current smoking (yes or no), current drinking (yes or no), and physical activity (MET-h/wk). According to the medians, WHR, WTR, and WNR were divided into high and low levels. The corresponding median value for WHR is 0.87 in women and 0.91 for men, for WTR is 1.66 in women and 1.74 for men, and for WNR is 2.48 in women and 2.34 in men

*, *P* < 0.05; HDL cholesterol, high-density lipoprotein cholesterol; WHR, waist-to-hip ratio; WTR, waist-to-thigh ratio; WNR, waist-to-neck ratio

The stability of body shapes was validated by the associations of body shapes with several major cardiometabolic disorders. In women, all body shapes were positively associated with metabolic syndrome and its components (elevated fasting blood glucose, high blood pressure, high triglycerides and low HDL cholesterol). These associations were strongest for the high WHR-high WTR-low WNR shape (Table 2). In men, low WHR-high WTR-high WNR shape and the higher WHR related shapes were associated with the higher risk of metabolic syndrome and its components. These associations were strongest for the high WHR-high WTR-low WNR shape (Table 2).

On consideration that WHR was the dominant determination indicator of insulin resistance and related cardiometabolic disorders. We further stratified the analysis by the WHR high- and low- levels (Table 3). In women, both in the high and low-WHR groups, those with high WTR shapes were significantly associated high risk of insulin resistance and related cardiometabolic disorders. In men, nearly all body shapes were not associated with insulin resistance and the related cardiometabolic disorders when stratified by WHR level (all *P* > 0.05, Table 3).

**Table 3:** Stratified analysis for associations body shape with insulin resistance and cardiometabolic disorders by WHR

Body shape	Insulin resistance	Metabolic syndrome	Elevated fasting blood glucose, including diabetes	High blood pressure	High triglycerides	Low HDL cholesterol
*Women*						
Low WHR-low WTR-low WNR	Ref	Ref	Ref	Ref	Ref	Ref
Low WHR-low WTR-high WNR	1.49 (0.89–1.75)	0.96 (0.70–1.33)	0.99 (0.74–1.34)	0.85 (0.65–1.12)	1.41 (1.08–1.85)*	1.09 (0.83–1.44)
Low WHR-high WTR-low WNR	2.10 (1.51–2.88)*	1.97 (1.47–2.65)*	1.77 (1.34–2.33)*	1.59 (1.18–2.14)*	1.57 (1.20–2.05)*	1.79 (1.37–2.33)*
Low WHR-high WTR-high WNR	2.09 (1.44–3.02)*	2.17 (1.53–3.06)*	2.32 (1.67–3.23)*	1.27 (0.88–1.84)	1.90 (1.38–2.63)*	1.15 (0.82–1.61)
High WHR-low WTR-low WNR	Ref	Ref	Ref	Ref	Ref	Ref
High WHR-low WTR-high WNR	0.95 (0.65–1.39)	1.01 (0.71–1.44)	1.05 (0.72–1.51)	1.15 (0.78–1.70)	1.00 (0.71–1.41)	0.83 (0.59–1.17)
High WHR-high WTR-low WNR	1.84 (1.28–2.64)*	1.39 (0.98–1.96)	1.79 (1.26–2.55)*	1.45 (0.97–2.16)	1.40 (1.00–1.95)	0.99 (0.71–1.39)
High WHR-high WTR-high WNR	1.41 (1.02–1.93)*	1.48 (1.10–2.00)*	1.49 (1.09–2.01)*	1.20 (0.86–1.67)	1.03 (0.78–1.38)	0.76 (0.58–1.03)
*Men*						
Low WHR-low WTR-low WNR	Ref	Ref	Ref	Ref	Ref	Ref
Low WHR-low WTR-high WNR	1.23 (0.76–1.98)	0.67 (0.41–1.10)	0.73 (0.51–1.05)	0.78 (0.55–1.11)	1.09 (0.75–1.59)	0.97 (0.62–1.53)
Low WHR-high WTR-low WNR	1.49 (0.91–2.44)	1.50 (0.96–2.34)	1.30 (0.91–1.85)	1.08 (0.74–1.58)	1.85 (1.27–2.69)*	1.30 (0.83–2.04)
Low WHR-high WTR-high WNR	2.41 (1.48–3.93)*	1.20 (0.73–2.00)	1.25 (0.84–1.87)	1.00 (0.65–1.54)	1.28 (0.82–1.98)	1.04 (0.61–1.77)
High WHR-low WTR-low WNR	Ref	Ref	Ref	Ref	Ref	Ref
High WHR-low WTR-high WNR	1.00 (0.62–1.61)	0.69 (0.44–1.09)	0.75 (0.40–1.14)	0.79 (0.49–1.27)	0.98 (0.64–1.51)	1.05 (0.66–1.65)
High WHR-high WTR-low WNR	1.42 (0.88–2.30)	1.07 (0.68–1.68)	0.99 (0.64–1.53)	1.56 (0.92–2.66)	1.00 (0.64–1.56)	0.99 (0.62–1.59)
High WHR-high WTR-high WNR	1.24 (0.83–1.87)	0.77 (0.53–1.14)	0.82 (0.57–1.18)	1.32 (0.86–2.01)	0.76 (0.53–1.10)	0.71 (0.47–1.05)

Data were presented as odds ratio (OR) and 95% confidence interval (CI). *P* values were calculated from multivariable logistic regression analysis. Adjusted age (years), body mass index (kg/m^2^), current smoking (yes or no), current drinking (yes or no), and physical activity (MET-h/wk). According to the medians, WHR, WTR, and WNR were divided into high and low levels. The corresponding median value for WHR is 0.87 in women and 0.91 for men, for WTR is 1.66 in women and 1.74 for men, and for WNR is 2.48 in women and 2.34 in men

*, *P* < 0.05; HDL cholesterol, high-density lipoprotein cholesterol; WHR, waist-to-hip ratio; WTR, waist-to-thigh ratio; WNR, waist-to-neck ratio

### Predictive values of anthropometric traits

In women, for insulin resistance, the addition of WHR, WTR, and WNR to a model containing BMI resulted in a change in the C statistic from 0.740 to 0.757, an NRI of 0.350, and an IDI 0.020 (all *P* < 0.001). For metabolic syndrome, the addition of WHR, WTR, and WNR to a model containing BMI resulted in a change in the C statistic from 0.743 to 0.787, an NRI of 0.368, and an IDI 0.034 (all *P* < 0.001). The combination of BMI, WHR, WTR, and WNR significantly increased C statistic, NRI and IDI for predicting the risk of insulin resistance and metabolic syndrome. In men, the inclusion of WHR, WTR, and WNR to a model containing BMI for predicting insulin resistance and metabolic syndrome significantly increased NRI and IDI (all *P* ≤ 0.04), except for C statistic (Additional file 1: Table S1).

We further compared the predictive value between “BMI + WHR + WNR + WTR” and “BMI + WHR” in evaluating insulin resistance and metabolic syndrome in total sample, BMI < 24 kg/m^2^ group, and BMI ≥ 24 kg/m^2^ group (Additional file 1: Table S3). We found “BMI + WHR + WNR + WTR” was significantly superior to “BMI + WHR” in predicting the risk of insulin resistance and metabolic syndrome in women with BMI less than 24 kg/m^2^, the ΔC statistic was 0.027 (0.012–0.042), 0.030 (0.010–0.049), respectively (all *P* < 0.003).

### Sensitivity analysis

We randomly selected 80% (n = 4492) individuals as training sample and performed logistic regression analyses of body shapes with insulin resistance and metabolic syndrome. The predictive value of BMI and BMI + WHR + WTR + WNR from the training sample were consistent with our main analysis. Additionally, in the training sample, we compared the sensitivity and specificity of BMI + WHR + WTR + WNR with BMI for diagnosing insulin resistance and metabolic syndrome. For insulin resistance, the inclusion of WHR, WTR, and WNR to a model containing BMI resulted in sensitivity changed from 60.1% to 64.7% in women, (63.1% to 64.5% in men); specificity changed from 75.8% to 72.3% in women (71.3% to 70.5% in men). For metabolic syndrome, the inclusion of WHR, WTR, and WNR to a model containing BMI resulted in sensitivity changed from 68.7% to 73.3% in women (57.8% to 60.4% in men), specificity changed from 66.8 to 63.4% in women (72.1% to 70.1% in men) (Additional file 1: Fig. S2).

We further compared the predictive value of multiple anthropometric traits in insulin resistance and metabolic syndrome in the test sample (the remaining 20%, n = 778). In women, for insulin resistance, the addition of weighted BMI + WHR + WTR + WNR to a model containing BMI resulted in C statistic changed from 0.713 to 0.729, sensitivity changed from 75.8% to 82.3%, specificity changed from 57.6% to 51.7%, an NRI of 0.579, and an IDI of 0.025 (all *P* < 0.002, Additional file 1: Fig. S2). For metabolic syndrome, when weighted BMI + WHR + WTR + WNR was added to a model with BMI, C-statistic changed from 0.724 to 0.760, sensitivity changed from 55.7% to 75.5%, specificity change from 78.0% to 66.0%, an NRI of 0.616, and an IDI of 0.054 (all* P* < 0.001, Additional file 1: Fig. S2). In men, the IDI significantly increased for insulin resistance and metabolic syndrome (all *P* < 0.05, Additional file 1: Fig. S2). These results indicated that the combination of multiple anthropometric traits can enhance the sensitivity for diagnosing cardiometabolic disorders.

## Discussion

In this cross-sectional study of 6240 community-dwelling Chinese adults, we defined 8 distinct body shapes based on sex-specific combinations of WHR, WTR and WNR, which differed in participants’ distribution and risk of insulin resistance and cardiometabolic disorders. In women, as compared to the low WHR-low WTR-low WNR shape, all the body shapes were significantly associated with a higher risk of IR and related cardiometabolic disorders. In men, as compared to low WHR-low WTR-low WNR shape, low WHR-high WTR-high WNR shape and the higher WHR related shapes were positively associated with insulin resistance and related cardiometabolic disorders. The risk of cardiometabolic disorders identified by multiple anthropometric traits independent of BMI and WHR, suggesting that the combination of multiple novel anthropometrics would benefit to identify additional cardiometabolic risk.

Emerging studies had illustrated that anthropometric traits were well-established predictors for insulin resistance, metabolic syndrome, type 2 diabetes and cardiovascular disease [7, 12, 14, 24]. For example, neck circumference and thinner thigh circumference were associated with an increased risk of peripheral arterial disease, cardiometabolic diseases and premature death [25, 26]. However, these studies only focused on solitary index rather than whole-body shape measured by multiple anthropometric traits, which couldn’t discriminate the difference of diseases susceptibility related to body shape diversity. Therefore, our study takes 3 ratios, WHR, WTR, and WNR, simultaneously into account to define 8 distinct body shapes, and evaluate their associations with risk of insulin resistance and cardiometabolic disorders. These body shapes showed distinct cardiometabolic risk in women.

In the current study, we compared the diagnostic performance of these anthropometric traits with routine clinical parameters (BMI). The results showed that the C-statistic improved slightly, the sensitivity improved, and the specificity decreased with the added anthropometric measures. C-statistic was widely used for the evaluation of risk prediction models. But it was insensitive as it often fails to detect improvements in the prediction that result from adding clinically relevant risk factors [27]. For example, adding novel indicators might increase the risk differences without improving discriminative ability when the C-statistic of the clinical prediction model is already high [27]. Although the C-statistic improved slightly in the present study, a clinically meaningful improvement in the NRI, IDI, and sensitivity was detected. In addition, in our study, the combination of BMI + WHR + WTR + WNR improved sensitivity without significantly reducing specificity. These results indicated that the combination of multiple anthropometric measures might increase the power to predict cardiometabolic risk, and was appropriate to large-scale epidemiological survey to identify more potential high-risk individuals. Our findings provide information for the development of better initial screening tools for cardiometabolic risk.

Mechanically, muscle mass loss of extremity circumference could influence insulin sensitivity, fat oxidation, and glucose metabolism and promote metabolic disorders [28, 29]. Neck fat accumulations could increase secretion of proinflammatory cytokine, elevated free fatty acid production, exacerbated systematic inflammation and impact glucose and lipid metabolism [30, 31]. These factors together exacerbated the risk of insulin resistance and related cardiometabolic disorders.

WHR was the dominant determination indicator of insulin resistance and related cardiometabolic disorders. In addition to WHR level, the WTR was the secondary determination indicator of insulin resistance and related cardiometabolic disorders in women. These results indicated that WTR may play a complementary role to WHR in predicting risk for cardiometabolic disorders. However, no significant associations were detected in men when stratified by the WHR level. Plausible explanations were biological differences between men and women, such as sex hormones metabolism, immune system responses, redistribution of body fat, muscle capacity and physical function [32, 33].

To the best of our knowledge, it is the first study to define refined body shapes by using multiple anthropometric traits simultaneously. The WHR, WTR, and WNR can represent the upper-, central- and lower- body fat distribution, and their measurements are convenient, simple, low cost, and harmless, which may benefit to refine define body shape and suit for large-scale population-based study. Besides, the well-defined community setting, fair sized sample volume, and desirable population homogeneity were a great foundation for current analysis. Several limitations should be acknowledged. Firstly, we could not establish a causal relationship between body shapes and the cardiometabolic risk. Due to the cross-sectional nature of this study, we only assessed the predictive ability of these anthropometric traits on the prevalence of cardiometabolic disorders. We could not obtain a “true predictive relationship” between body shape and future metabolic risk. The prospective follow-up studies are warranted to further verify the results in the current study. Secondly, the onset of cardiometabolic diseases is insidious, mostly occurring aged 40 years or above. Body composition changed with aging. Our study was conducted in middle and elderly adults, and it should be cautious to interpret the results to the youngers. Thirdly, adopting multiple anthropometric traits to define a comprehensive body shape is more complicated than BMI and WHR in clinical practice. However, a recent study indicated that the combination of multiple indicators would provide a powerful tool to predict metabolic disorders [34]. Our study presented a whole picture of the body shape defined by multiple anthropometric parameters other than BMI, which could capture additional cardiometabolic risk and may provide a useful value in disease screening. Fourthly, the cut-off value of WHR, WTR, and WNR in distinguishing cardiometabolic risk was just based on a statistical significance, it is far to be used as a diagnostic standard. The WHR, WTR, and WNR cut-off values should be further verified in larger prospective cohorts in different age or ethnicity groups.

## Conclusions

In conclusion, we defined 8 distinct body shapes by taking WHR, WTR, and WNR, simultaneously into account in a Chinese community-based cohort. The combination of WHR, WTR, and WNR was significantly associated with a higher risk of insulin resistance and cardiometabolic disorders independent of BMI or WHR. The defined body shapes do provide additional cardiometabolic risk assessment over BMI and WHR. Our findings provided a useful, simple, and available method to discriminate parts of individual variation in the cardiometabolic risk.

## Supplementary Information

**Additional file 1: Figure S1**. Feature selection. A: Automated feature selection. It was performed by using the information gain attribute ranking method on the open-source Waikato Environment for Knowledge Analysis platform. Information gain ranking was used to evaluate the worth of each variable (usually the clinical indicator) by measuring the entropy gain to the outcome. The greater the information gain a clinical indicator has, the more important the indicator is in the classification process. The model was built with logistic regression analysis based on the data set. The regression coefficient of each significant variable was regarded as the contribution level. B: Entropy gain for each indicator. It illuminates the information gain and ranks the attributes of each variable from the top to the bottom. Three indicators (WHR, WNR and WTR) were chosen to construct body shapes. Abbreviation: WHR, waist-to-hip ratio; WTR, waist-to-thigh ratio; WNR, waist-to-neck ratio; NTR, neck-to-thigh ratio; NHR, neck-to-hip ratio; THR, thigh-to-hip ratio. **Figure S2**. Predictive values of anthropometric traits in training sample and test sample. The predictive value of BMI and BMI+WHR+WTR+WNR for diagnosing insulin resistance and metabolic syndrome in training sample (A, B). The predictive value of BMI and BMI+WHR+WTR+WNR for diagnosing insulin resistance and metabolic syndrome in testing sample (C, D). Data are C statistic, ΔC statistic, IDI and NRI, 95% confidence intervals, sensitivity, specificity. P values were from logistic analysis. Abbreviation: NRI, net reclassification improvement; IDI, integrated dis-crimination improvement; BMI, body mass index; WHR, waist-to-hip circumference ratio; WTR, waist-to-thigh circumference ratio; WNR, waist-to-neck circumference ratio. * indicated P value <0.05. **Table S1**. Predictive values of anthropometric traits in total sample. **Table S2**. Association of body shape with risk of cardiovascular diseases in training sample. **Table S3**. Predictive values of anthropometric traits in stratification analysis.

## Data Availability

All data generated or analyzed during this study are included in this published article (and its Additional information files).

## Abbreviations

BMI: Body mass index
IR: Insulin resistance
WHR: Waist-to-hip ratio
WTR: Waist-to-thigh ratio
WNR: Waist-to-neck ratio
OGTT: Oral glucose tolerance tests
HDL: Cholesterol, high-density lipoprotein cholesterol
LDL: Cholesterol, low-density lipoprotein cholesterol
HOMA-IR: Homeostasis model assessment of insulin resistance
NRI: Net reclassification improvement
IDI: Integrated discrimination improvement
