# Supplementary material for: Associations of body shapes with insulin resistance and cardiometabolic risk in middle-aged and elderly Chinese
Source: Nutr Metab (Lond). 2021 Dec 7;18:103. doi: 10.1186/s12986-021-00629-1 (PMC8650554; doi:10.1186/s12986-021-00629-1)
Supplement: Supplementary file 1 — Additional file 1: Figure S1. Feature selection. A: Automated feature selection. It was performed by using the information gain attribute ranking method on the open-source Waikato Environment for Knowledge Analysis platform. Information gain ranking was used to evaluate the worth of each variable (usually the clinical indicator) by measuring the entropy gain to the outcome. The greater the information gain a clinical indicator has, the more important the indicator is in the classification process. The model was built with logistic regression analysis based on the data set. The regression coefficient of each significant variable was regarded as the contribution level. B: Entropy gain for each indicator. It illuminates the information gain and ranks the attributes of each variable from the top to the bottom. Three indicators (WHR, WNR and WTR) were chosen to construct body shapes. Abbreviation: WHR, waist-to-hip ratio; WTR, waist-to-thigh ratio; WNR, waist-to-neck ratio; NTR, neck-to-thigh ratio; NHR, neck-to-hip ratio; THR, thigh-to-hip ratio. Figure S2. Predictive values of anthropometric traits in training sample and test sample. The predictive value of BMI and BMI+WHR+WTR+WNR for diagnosing insulin resistance and metabolic syndrome in training sample (A, B). The predictive value of BMI and BMI+WHR+WTR+WNR for diagnosing insulin resistance and metabolic syndrome in testing sample (C, D). Data are C statistic, ΔC statistic, IDI and NRI, 95% confidence intervals, sensitivity, specificity. P values were from logistic analysis. Abbreviation: NRI, net reclassification improvement; IDI, integrated dis-crimination improvement; BMI, body mass index; WHR, waist-to-hip circumference ratio; WTR, waist-to-thigh circumference ratio; WNR, waist-to-neck circumference ratio. * indicated P value <0.05. Table S1. Predictive values of anthropometric traits in total sample. Table S2. Association of body shape with risk of cardiovascular diseases in training sample. Table S3. Predictiv [file 12986_2021_629_MOESM1_ESM.docx]

**SUPPLEMENTAL MATERIALS**

**TITLE: Associations of Body Shapes with Insulin Resistance and Cardiometabolic Risk in Middle-aged and Elderly Chinese**

**AUTHORS: Yulin Zhou, Yanan Hou, Jiali Xiang, et al.**

**Figure legends**

**Supplemental Figure 1.** Feature selection. A: Automated feature selection. It was performed by using the information gain attribute ranking method on the open-source Waikato Environment for Knowledge Analysis platform. Information gain ranking was used to evaluate the worth of each variable (usually the clinical indicator) by measuring the entropy gain to the outcome. The greater the information gain a clinical indicator has, the more important the indicator is in the classification process. The model was built with logistic regression analyses based on the data set. The regression coefficient of each significant variable was regarded as the contribution level. B: Entropy gain for each indicator. It illuminates the information gain and ranks the attributes of each variable from the top to the bottom. Three indicators (WHR, WNR and WTR) were chosen to construct body shapes. Abbreviation: WHR, waist-to-hip ratio; WTR, waist-to-thigh ratio; WNR, waist-to-neck ratio; NTR, neck-to-thigh ratio; NHR, neck-to-hip ratio; THR, thigh-to-hip ratio.

**Supplemental Figure 2.** Predictive values of anthropometric traits in training sample

and test sample. The predictive value of BMI and BMI+WHR+WTR+WNR for diagnosing insulin resistance and metabolic syndrome in training sample (A, B). The predictive value of BMI and BMI+WHR+WTR+WNR for diagnosing insulin resistance and metabolic syndrome in testing sample (C, D). Data are C statistic, ΔC statistic, IDI and NRI, 95% confidence intervals, sensitivity, specificity. *P* values were from logistic analysis. Abbreviation: NRI, net reclassiﬁcation improvement; IDI, integrated dis-crimination improvement; BMI, body mass index; WHR, waist-to-hip circumference ratio; WTR, waist-to-thigh ratio; WNR, waist-to-neck ratio. * indicated P value <0.05.

**Supplemental Figure 1.** Feature selection.


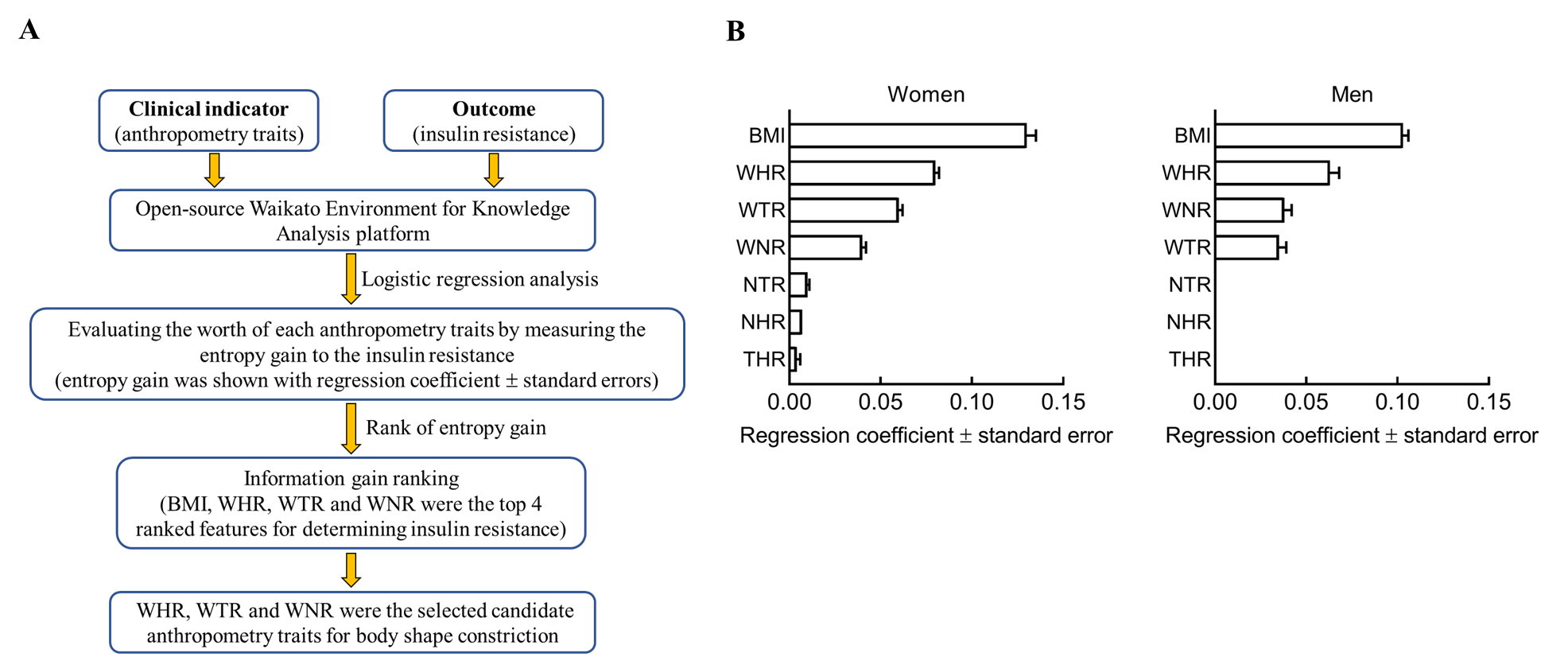


**Supplemental Figure 2.** Predictive values of anthropometric traits in training sample

and test sample.


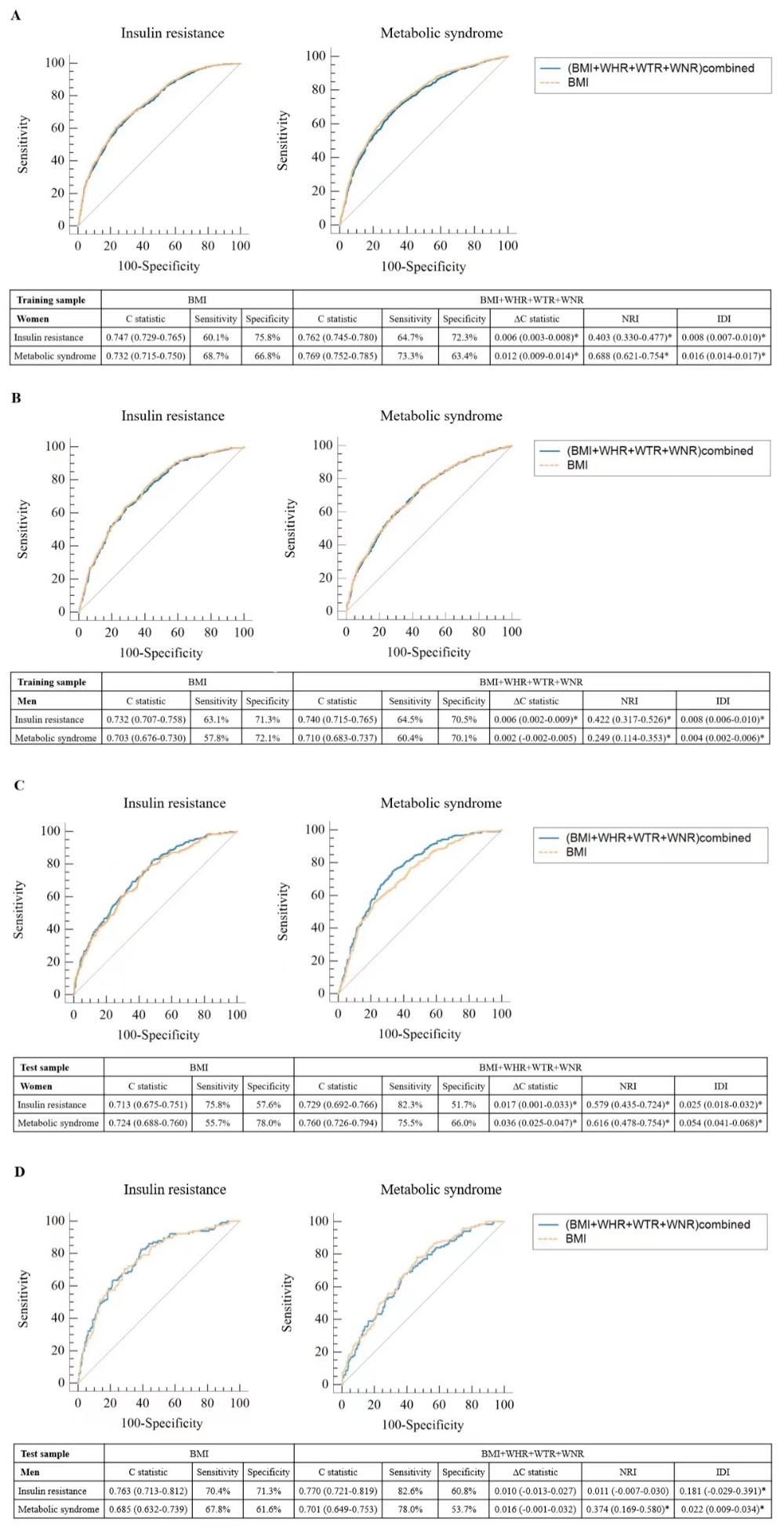


**Supplemental Table 1.** Predictive values of anthropometric traits in total sample.

|  | BMI | BMI+WHR+WTR+WNR | | | |
| --- | --- | --- | --- | --- | --- |
|  | C statistic | C statistic | ΔC statistic | NRI | IDI |
| **Women** |  |  |  |  |  |
| Insulin resistance | 0.740 (0.724-0.756) | 0.757 (0.742-0.773) | 0.017 (0.010-0.024)* | 0.350 (0.284-0.416)* | 0.020 (0.015-0.025)* |
| Metabolic syndrome | 0.743 (0.728-0.759) | 0.787 (0.773-0.801) | 0.012 (0.009-0.022)* | 0.368 (0.302-0.434)* | 0.034 (0.027-0.042)* |
| **Men** |  |  |  |  |  |
| Insulin resistance | 0.739 (0.716-0.761) | 0.747 (0.725-0.770) | 0.009 (-0.001-0.019) | 0.279 (0.184-0.373)* | 0.016 (0.009-0.023)* |
| Metabolic syndrome | 0.774 (0.754-0.794) | 0.795 (0.776-0.814) | 0.096 (0.000-0.019) | 0.259 (0.166-0.353)* | 0.013 (0.007-0.018)* |

Data are C statistic, ΔC statistic, IDI and NRI, 95% confidence intervals. P values were from logistic analysis. Abbreviation: NRI, net reclassiﬁcation improvement; IDI, integrated dis-crimination improvement; BMI, body mass index; WHR, waist-to-hip ratio; WTR, waist-to-thigh ratio; WNR, waist-to-neck ratio. * indicated P value <0.04

**Supplemental Table 2.** Association of body shape with risk of cardiovascular diseases in training sample

|  | **Training sample** | |
| --- | --- | --- |
| **Body shape** | Insulin resistance | Metabolic syndrome |
| **Women (n= 2694)** |  |  |
| Low WHR-low WTR-low WNR | Ref. | Ref. |
| Low WHR-low WTR-high WNR | 1.17 (0.79-1.74) | 0.89 (0.61-1.29) |
| Low WHR-high WTR-low WNR | 2.06 (1.44-2.95)* | 1.91 (1.37-2.67)* |
| Low WHR-high WTR-high WNR | 2.35 (1.58-3.49)* | 2.32 (1.60-3.37)* |
| High WHR-low WTR-low WNR | 1.87 (1.28-2.75)* | 2.52 (1.78-3.56)* |
| High WHR-low WTR-high WNR | 2.18 (1.56-3.05)* | 2.94 (2.15 -4.03)* |
| High WHR-high WTR-low WNR | 4.03 (2.93-5.54)* | 3.87 (2.85-5.25)* |
| High WHR-high WTR-high WNR | 3.00 (2.32-3.89)* | 3.97 (3.12-5.04)* |
| **Men (n= 1818)** |  |  |
| Low WHR-low WTR-low WNR | Ref. | Ref. |
| Low WHR-low WTR-high WNR | 1.38 (0.81-2.34) | 0.57 (0.31-1.02) |
| Low WHR-high WTR-low WNR | 1.44 (0.82-2.51) | 1.54 (0.94-2.51) |
| Low WHR-high WTR-high WNR | 2.27 (1.31 -3.95)* | 1.20 (0.69-2.10) |
| High WHR-low WTR-low WNR | 1.78 (1.06-2.98)* | 2.07 (1.31-3.28)* |
| High WHR-low WTR-high WNR | 2.24 (1.40-3.58)* | 1.78 (1.14-2.77)* |
| High WHR-high WTR-low WNR | 3.30 (2.07-5.28)* | 2.36 (1.52-3.67)* |
| High WHR-high WTR-high WR | 2.62 (1.79-3.82)* | 1.79 (1.26-2.53)* |

Data were presented as odds ratio (OR) and 95% confidence interval (CI). *P* values were calculated from multivariable logistic regression analysis. Adjusted age (years), body mass index (kg/m^2^), current smoking (yes or no), current drinking (yes or no), and physical activity (MET-h/wk). According to the medians, WHR, WTR, and WNR were divided into high and low levels. The corresponding median value for WHR is 0.87 in women and 0.91 for men, for WTR is 1.66 in women and 1.74 for men, and for WNR is 2.48 in women and 2.34 in men. *: *P* < 0.05; Abbreviation: WHR, waist-to-hip ratio; WTR, waist-to-thigh ratio; WNR, waist-to-neck ratio.

**Supplemental Table 3.** Predictive values of anthropometric traits in stratification analysis**.**

|  | BMI+WHR | BMI+WHR+WNR+WTR | |
| --- | --- | --- | --- |
|  | C statistic | C statistic | ΔC statistic |
| **Total sample** |  |  |  |
| **Women** |  |  |  |
| Insulin resistance | 0.747 (0.731-0.763) | 0.757 (0.742-0.773) | 0.011 (0.005-0.017)* |
| Metabolic syndrome | 0.764 (0.749-0.779) | 0.768 (0.753-0.783) | 0.004 (-0.001-0.010) |
| **Men** |  |  |  |
| Insulin resistance | 0.747 (0.725-0.770) | 0.747 (0.725-0.770) | / |
| Metabolic syndrome | 0.709 (0.685-0.733) | 0.709 (0.685-0.733) | / |
| **BMI <24 kg/m^2^** |  |  |  |
| **Women** |  |  |  |
| Insulin resistance | 0.690 (0.659-0.721) | 0.717 (0.686-0.748) | 0.027 (0.012-0.042)* |
| Metabolic syndrome | 0.656 (0.626-0.687) | 0.686 (0.656-0.715) | 0.030 (0.010-0.049)* |
| **Men** |  |  |  |
| Insulin resistance | 0.646 (0.577-0.715) | 0.646 (0.583-0.709) | -0.0002 (-0.048-0.048) |
| Metabolic syndrome | 0.625 (0.570-0.681) | 0.628 (0.574-0.682) | 0.003 (-0.019-0.024) |
| **BMI≥24 kg/m^2^** |  |  |  |
| **Women** |  |  |  |
| Insulin resistance | 0.696 (0.674-0.718) | 0.700 (0.678-0.722) | 0.004 (-0.006-0.014) |
| Metabolic syndrome | 0.708 (0.687-0.730) | 0.713 (0.692-0.735) | 0.005 (-0.001-0.011) |
| **Men** |  |  |  |
| Insulin resistance | 0.665 (0.635-0.694) | 0.664 (0.635-0.694) | -0.0002 (-0.010-0.010) |
| Metabolic syndrome | 0.656 (0.626-0.686) | 0.656 (0.626-0.686) | -0.0002 (-0.010-0.009) |

Data are C statistic, ΔC statistic, 95% confidence intervals. P values were from logistic analysis. Abbreviation: BMI, body mass index; WHR, waist-to-hip ratio; WTR, waist-to-thigh ratio; WNR, waist-to-neck ratio. * indicated P value <0.003
